# Personal interventions to reduce air pollution exposure in a representative sample of Poles aged 18–64 years

**DOI:** 10.3389/fpubh.2025.1656587

**Published:** 2025-11-26

**Authors:** Radosław Sierpiński, Mateusz Jankowski, Filip Raciborski

**Affiliations:** 1 Faculty of Medicine, Collegium Medicum, Cardinal Stefan Wyszynski University, Warsaw, Poland; 2 Department of Population Health, School of Public Health, Centre of Postgraduate Medical Education, Warsaw, Poland; 3 Department of Prevention of Environmental Hazards, Allergology and Immunology, Faculty of Health Sciences, Medical University of Warsaw, Warsaw, Poland

**Keywords:** air pollution, prevention, personal intervention, air quality, environmental health

## Abstract

**Introduction:**

Exposure to air pollution is a major environmental hazards to human health. This study aimed to assess personal interventions to reduce air pollution exposure in a representative sample of Poles aged 18–64 years, as well as to identify factors associated with these personal interventions.

**Methods:**

This is a secondary analysis of data from the nationwide cross-sectional survey carried out in December 2024 in a representative sample of adults in Poland aged 18–64 years. Questions on four personal interventions to reduce air pollution exposure were analyzed.

**Results:**

In the analyzed population (*n* = 5,006), the percentage of women was 49.9%, average age of 41.8 (SD = 12.59). Among the respondents, 18.2% reported closing windows at home to protect against air pollution, and 16.1% reported using air purifiers at home. Avoiding outdoor walking during high-pollution periods and monitoring air-quality alerts were reported by 12.7 and 11.8%, respectively. Males had a 15% higher odds of using at least one intervention (OR = 1.15; 95%CI: 1.02–1.31). Adults aged 25–34 showed a 48% higher odds of using at least one personal intervention compared to people aged 45–64 (OR = 1.48; 95%CI: 1.24–1.78). Residents of the largest cities (>500,000 inhabitants) were 46% more likely to implement personal interventions to reduce air pollution exposure in comparison to residents of rural areas (OR = 1.46; 95%CI: 1.19–1.78). Having children aged 4–12 years (*p* < 0.05) increased the odds of implementation of personal intervention in comparison to people without children of this age. Individuals with frequent infections (≥5 per year) had 46% higher odds than those who reported not getting sick (OR = 1.46; 95%CI: 1.07–2.00).

**Conclusion:**

Adults in Poland show low uptake of personal measures to reduce air pollution exposure. Socio-demographic differences in the implementation of a personal intervention to reduce air pollution were observed, with particular emphasis on gender, age, place of residence, having children, and economic status.

## 1 Introduction

Exposure to air pollution is one of the most important environmental hazards to human health (1, 2). The World Health Organization (WHO) defines air pollution as „contamination of the indoor or outdoor environment by any chemical, physical or biological agent that modifies the natural characteristics of the atmosphere” (3). Particulate matter, carbon monoxide, ozone, nitrogen dioxide, and sulfur dioxide are major pollutants (3). Short-term and long-term exposure to air pollution is associated with an increased risk of diseases, including cardiovascular diseases, diseases of the respiratory system, and cancers (1, 2, 4). The WHO estimates that exposure to air pollution is associated with 6.7 million premature deaths annually, including 4.2 million deaths from ambient air (outdoor) pollution and 3.2 million from indoor (household) air pollution (3).

There are multiple sources of air pollution. However, transportation, residential heating, industrial production, and agriculture are the major sources of outdoor air pollution (1, 2, 5). Daily home activities such as cooking, smoking, and the use of consumer products are major sources of indoor air pollution (6). Emissions from building materials also contribute to the indoor air pollution (6). Outdoor air pollution can enter homes primarily through open windows and doors, but also through cracks in walls, doors, and window sealants (7).

Given the high burden of diseases attributed to air pollution, numerous public health interventions – targeting both populations and individuals, have been implemented globally (1, 3, 8, 9). At the national level, interventions aim to improve air quality by reducing emissions through legal and organizational measures (e.g., bans on burning specific fuels, promotion of electric mobility, and development of construction standards) (8–10). Legislative approaches include incentive, supportive and punitive policies (9). Beyond population-level interventions, the scientific literature increasingly highlights the role of individual- and household-level interventions to reduce personal exposure to air pollution (8, 10). Such interventions include wearing respirators, using indoor portable air purifiers (cleaners), avoiding polluted places, and limiting outdoor activities or exercising during episodes of high air pollution (11, 12).

Poland is the fifth largest European Union (EU) country, with a population of 38 million (13). A World Bank Group report estimated 36 of the 50 most polluted cities in the EU were in Poland (14). Exposure to air pollution is therefore a significant and urgent public health problem in Poland. However, there is a research gap regarding public knowledge and attitudes toward air pollution, particularly a lack of up-to-date, nationally representative data.

This study aimed to assess personal interventions to reduce air pollution exposure in a representative sample of Poles aged 18–64 years, as well as to identify factors associated with these personal interventions.

## 2 Materials and methods

### 2.1 Study design and data source

This is a secondary data analysis of data from the nationwide cross-sectional survey called “Health prevention and health inequalities” (15) and performed in December 2024 by the National Centre for Health Policy and Health Inequalities of the Cardinal Stefan Wyszynski University. The original dataset was generated under the contract with the Polish Ministry of Education and Science (Agreement no. MEiN/2023/DPI/2717 of 13/10/2023) and adhered to a data availability policy that included free-of-charge sharing for scientific purposes.

An official request was submitted to the National Centre for Health Policy and Health Inequalities, and a database covering a raw dataset of 12 questions was obtained.

### 2.2 Population

Original data came from a cross-sectional survey carried out in a representative sample of adults in Poland aged 18–64 years (15, 16). Data were collected by a dedicated public opinion poll company (ARC Rynek i Opinia) on behalf of the National Centre for Health Policy and Health Inequalities (17). The study questionnaire was available online via a dedicated website and IT system. The stratification model included: gender, age, size of the place of residence, as well as level of education. National demographic data published by the Statistics of Poland were used to set a demographic structure of the study sample (13). Participation in the cross-sectional study was voluntary and anonymous. Informed consent forms were collected. The study protocol was reviewed and approved by the Ethical Committee at the Medical University of Warsaw (decision number: AKBE/56/2025).

### 2.3 Measures

Personal interventions to reduce air pollution exposure were identified based on the following question: Which of the following personal interventions to reduce air pollution exposure do you perform: I use air purifiers at home all year round; I use air purifiers at home during the heating season; I close windows to reduce air pollution (e.g., due to traffic or smog); I avoid walking outside during periods of high air pollution; I monitor air quality alerts; None of the above, each intervention with two possible answers: yes or no. For this article, responses to the questions “I use air purifiers at home all year round” and “I use air purifiers at home during the heating season” were combined into one category.

Moreover, responses to 11 questions on personal characteristics were analyzed (Supplementary File S1).

### 2.4 Statistical analysis

Records received from the Centre for Health Policy and Health Inequalities were used to prepare datasets used for statistical analysis (15). Data were analyzed with IBM SPSS Statistics version 29.0 (IBM Corp., Armonk, NY, United States) statistical software. Demographic weight was applied. Descriptive data were presented with frequencies and proportions. The chi-squared test was used to analyze differences between qualitative variables. The multivariable logistic regression model was prepared to identify factors associated with the implementation of at least one personal intervention to reduce air pollution exposure. All 4 analyzed personal intervention to reduce air pollution exposure were combined into one variable. The model quality assessment was calculated based on the Cox and Snell R-square and the Nagelkerke *R*-square values. Odds ratios (OR) and 95-percent confidence intervals (95%CI) were used to present strengths of associations. The statistical significance level was set at *p* < 0.05.

## 3 Results

In the analyzed population (*n* = 5,006) the percentage of women was 49.9%. The age range of the respondents was 18–64 years, with an average age of 41.8 (SD = 12.59), median of 42 years. Overall, 30.3% had higher education. Rural residents constituted 40.6% of the respondents. In the study group, 11.8% had children up to 4 years of age, and 22.5% between 5 and 12 years of age. Over 75% were employed (full-time 57.2% and part-time or occasional 18.0%). Detailed data are presented in Table 1.

**Table 1:** Characteristics of the analyzed population (*n* = 5,006).

	*n*	%
Total	5,006	100.0
Gender		
Male	2,506	50.1
Female	2,500	49.9
Age [years]		
18–24	541	10.8
25–34	990	19.8
35–44	1,326	26.5
45–64	2,149	42.9
Educational level		
Primary or vocational	1,593	31.8
Secondary	1896	37.9
Higher	1,518	30.3
Place of residence		
Rural area	2035	40.6
City < 100,000 residents	1,581	31.6
City 100,000–499,000 residents	789	15.8
City ≥ 500,000 residents	601	12.0
Having children under 4 years of age		
No	4,416	88.2
Yes	590	11.8
Having children aged 5–8 years		
No	4,330	86.5
Yes	676	13.5
Having children aged 9–12 years		
No	4,342	86.7
Yes	664	13.3
Having children aged 13–17 years		
No	4,067	81.3
Yes	939	18.7
Having job (currently working)		
Yes, full-time job	2,862	57.2
Yes, part-time job	902	18.0
No	1,242	24.8
Self-assessment of household financial situation		
We have enough money for everything and we are still saving for the future	999	19.9
We have enough money for everything without any special sacrifices but we do not save for the future	977	19.5
We live frugally and thanks to that we have enough money for everything	1805	36.0
We live very frugally in order to save money for more serious purchases	679	13.6
We only have enough money for basic needs or there is not enough money even for the cheapest food	547	10.9

**Figure 1:**
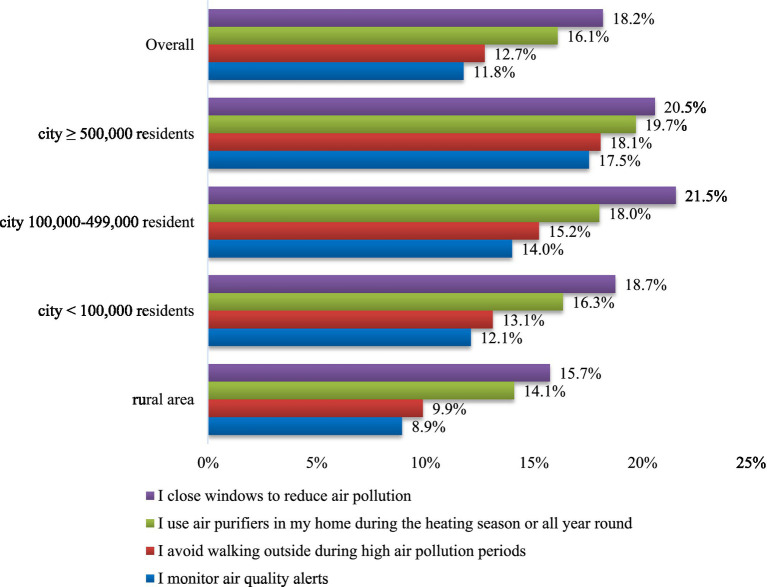
Personal interventions to protect against air pollution by place of residence.

In the analyzed population (*n* = 5,006), 18.2% reported closing windows at home to protect against air pollution (Figure 1). The second most common intervention was using an air purifier at home (all year round or during the heating season), reported by 16.1%. The other two methods: avoiding outdoor walking during periods of high air pollution and monitoring air quality alerts were reported by 12.7 and 11.8%, respectively. These interventions were less frequent among rural than urban residents (e.g., closing windows 15.7% vs. 19.9%, *p* < 0.001; using air purifiers 14.1% vs. 17.4%, *p* < 0.01). Detailed data are presented in Figure 1.

Gender differences (Table 2) were observed only for closing windows – 19.4% of men used this method compared to 16.9% of women (*p* < 0.05). Age had a statistically significant effect on the results obtained in all the analyzed methods. The group that most often declared using at least 1 of the 4 analyzed methods were people aged 25–34. Among them, this percentage was 49.7%. For comparison, the lowest percentage was among people aged 45–64 (34.1%; *p* < 0.001). Among respondents with higher education, 48.2% used ≥1 intervention versus 35.8% among those with primary or vocational education (*p* < 0.001). The younger the children in the household, the greater the likelihood of implementing interventions: in households with children up to 4 years, 52.1% used ≥1 method versus 40.0% among those without children in this age group (*p* < 0.001). Detailed data are presented in Table 2.

**Table 2:** Personal interventions to reduce air pollution exposure by place of residence by socio-demographic variables (*n* = 5,006).

	*n*	Air purifier at home	p	Closing windows at home	*p*	Avoiding walks outside	*p*	Monitoring air quality alerts	*p*	At least one intervention	*p*
Overall	5,006	16.1%		18.2%		12.7%		11.8%		41.4%	
Gender											
Male	2,506	15.7%	0.465	19.4%	<0.05	12.4%	0.581	11.4%	0.429	41.4%	0.909
Female	2,500	16.5%		16.9%		13.0%		12.1%		41.5%	
Age											
18–24	541	13.2%	<0.001	21.3%	<0.001	10.9%	<0.001	11.8%	<0.05	45.5%	<0.001
25–34	990	19.6%		22.6%		15.7%		14.2%		49.7%	
35–44	1,326	20.3%		19.6%		14.3%		11.8%		45.6%	
45–64	2,149	12.5%		14.4%		10.8%		10.6%		34.1%	
Educational level											
Primary or vocational	1,593	16.1%	0.113	14.0%	<0.001	8.2%	<0.001	8.4%	<0.001	35.8%	<0.001
Secondary	1896	14.9%		19.2%		12.2%		11.1%		40.8%	
Higher	1,518	17.5%		21.2%		18.1%		16.1%		48.1%	
Place of residence											
Rural area	2035	14.1%	<0.01	15.7%	<0.01	9.9%	<0.001	8.9%	<0.001	35.6%	<0.001
City < 100,000 residents	1,581	16.3%		18.7%		13.1%		12.1%		43.3%	
City 100,000–499,000 residents	789	18.0%		21.5%		15.2%		14.0%		47.5%	
City ≥ 500,000 residents	601	19.7%		20.5%		18.1%		17.5%		48.2%	
Having children under 4 years of age											
No	4,416	14.6%	<0.001	17.7%	<0.05	12.2%	<0.01	11.5%	0.196	40.0%	<0.001
Yes	590	27.3%		21.5%		16.3%		13.4%		52.1%	
Having children aged 5–8 years											
No	4,330	15.1%	<0.001	17.8%	0.086	12.6%	0.620	11.4%	0.083	40.4%	<0.001
Yes	676	22.1%		20.6%		13.3%		13.8%		48.4%	
Having children aged 9–12 years											
No	4,342	15.5%	<0.05	18.1%	0.705	12.7%	0.950	12.0%	0.219	40.8%	p < 0.05
Yes	664	19.6%		18.7%		12.7%		10.3%		45.3%	
Having children aged 13–17 years											
No	4,067	16.3%	0.301	18.9%	<0.01	13.2%	<0.05	12.0%	0.369	42.1%	0.051
Yes	939	14.9%		15.2%		10.7%		10.9%		38.6%	
Having job (currently working)											
Yes, full-time job	2,862	17.3%	<0.01	18.3%	<0.001	12.1%	<0.001	11.2%	<0.001	41.5%	<0.001
Yes, part-time job	902	16.6%		25.4%		18.7%		16.1%		52.6%	
No	1,242	12.9%		12.7%		9.8%		9.9%		33.3%	
Self-assessment of household financial situation											
We have enough money for everything and we are still saving for the future	999	22.0%	<0.001	21.2%	<0.01	15.6%	<0.01	15.9%	<0.001	47.5%	<0.001
We have enough money for everything without any special sacrifices but we do not save for the future	977	17.2%		17.9%		12.8%		11.0%		43.6%	
We live frugally and thanks to that we have enough money for everything	1805	14.1%		16.5%		12.4%		10.8%		39.6%	
We live very frugally in order to save money for more serious purchases	679	13.0%		21.1%		12.5%		11.9%		41.5%	
We only have enough money for basic needs or there is not enough money even for the cheapest food	547	13.5%		14.6%		8.6%		8.8%		32.6%	

People who rated their health better than their peers were also most likely to report using air purifiers (21.4%; *p* < 0.001) and monitoring air quality alerts (14.0%; *p* < 0.05) (Table 3). In this group, 46.8% used ≥1 of the four interventions (*p* < 0.001). Those reporting their health as decidedly worse than peers used these methods least often – 12.4, 11.2, and 34.7%, respectively. Individuals who reported getting sick ≥5 times a year were the most likely to use air purifiers (20.9%; *p* < 0.001), close windows (23.1%; *p* < 0.001), and avoid walking (15.5%; *p* < 0.01). In general, 50.8% of this group implemented ≥1 intervention (Table 3). Similarly, respondents with ≥3 chronic conditions most often closed windows (22.0%; *p* < 0.05), avoided walks (16.5%; *p* < 0.01), and monitored air quality alerts (19.9%; *p* < 0.001). In general, 46.1% of this group implemented ≥1 intervention (*p* < 0.05). Detailed results are presented in Table 3.

**Table 3:** Personal interventions to reduce air pollution exposure by self-reported health status (*n* = 5,006).

	*n*	Air purifier at home	*p*	Closing windows at home	*p*	Avoiding walks outside	*p*	Monitoring air quality alerts	*p*	At least one intervention	*p*
Self-assessment of health status compared to peers											
Definitely better or a little better	1,343	21.4%	<0.001	19.4%	0.465	14.7%	0.062	14.0%	<0.05	46.8%	<0.001
The same or hard to say	2,323	13.6%		17.7%		11.7%		10.7%		39.2%	
A little worse	1,003	16.0%		18.1%		12.5%		11.5%		41.7%	
Definitely worse	337	12.4%		16.2%		12.7%		11.2%		34.7%	
On average. How often do you get sick or catch a cold during the year?											
I never get sick	460	16.8%	<0.001	13.9%	<0.001	10.0%	<0.01	7.3%	<0.01	33.8%	<0.001
1–2 times a year	3,125	13.9%		16.8%		11.9%		11.9%		38.7%	
3–4 times a year	1,057	20.6%		22.3%		15.2%		14.1%		49.7%	
5 times a year or more	364	20.9%		23.1%		15.5%		9.3%		50.8%	
Number of chronic diseases											
No disease	2,359	15.1%	0.098	17.2%	<0.05	11.1%	<0.01	9.7%	<0.001	39.9%	<0.05
1 disease	1,281	16.5%		17.9%		13.5%		10.8%		42.2%	
2 diseases	699	15.9%		18.4%		13.3%		12.5%		40.7%	
3 or more	667	19.0%		22.0%		16.5%		19.9%		46.1%	

Among respondents who do not seek health information, significantly lower frequencies were observed for all interventions compared with those who seek health information (Table 4): air purifiers 10.1%. 18.6%, *p* < 0.001; closing windows 8.1% vs. 22.4%, *p* < 0.001; avoided walking during periods of high air pollution 5.2% vs. 15.9%, *p* < 0.001; and monitoring alerts 3.7% vs. 15.1%, *p* < 0.001. Overall, 21.9% in this group used ≥1 intervention (vs. 49.6%; *p* < 0.001). Data by individual sources of health information are presented in Table 4.

**Table 4:** Personal interventions to reduce air pollution exposure by different sources of health information (*n* = 5,006).

	*n*	Air purifier at home	*p*	Closing windows at home	*p*	Avoiding walks outside	*p*	Monitoring air quality alerts	*p*	At least one intervention	*p*
I am not looking for health information											
No	3,531	18.6%	<0.001	22.4%	<0.001	15.9%	<0.001	15.1%	<0.001	49.6%	<0.001
Yes	1,475	10.1%		8.1%		5.2%		3.7%		21.9%	
On the websites of public institutions (e.g., Ministry of Health or WHO)											
No	4,490	14.9%	<0.001	17.0%	<0.001	11.5%	<0.001	9.9%	<0.001	38.9%	<0.001
Yes	516	25.9%		28.4%		23.8%		27.8%		63.7%	
On dedicated health websites (e.g., medonet. Abczdrowie)											
No	3,405	15.3%	<0.05	15.5%	<0.001	10.1%	<0.001	7.8%	<0.001	37.4%	<0.001
Yes	1,601	17.8%		23.9%		18.4%		20.2%		49.9%	
On other websites or forums											
No	3,871	15.9%	0.435	17.3%	<0.01	12.2%	0.061	10.3%	<0.001	40.2%	<0.01
Yes	1,135	16.8%		21.2%		14.4%		16.7%		45.6%	
On social media (e.g., Facebook, Instagram, TiKTok)											
No	4,128	14.7%	<0.001	16.3%	<0.001	11.3%	<0.001	10.3%	<0.001	38.2%	<0.001
Yes	878	22.4%		26.8%		19.4%		18.6%		56.6%	
On YouTube or similar websites											
No	4,090	15.1%	<0.001	16.4%	<0.001	11.6%	<0.001	10.3%	<0.001	38.6%	<0.001
Yes	916	20.6%		26.1%		17.7%		18.2%		54.1%	
Among friends (but not medical personnel)											
No	4,333	15.1%	<0.001	16.8%	<0.001	11.8%	<0.001	10.5%	<0.001	39.6%	<0.001
Yes	673	22.6%		26.9%		18.4%		19.8%		53.2%	
Among family members (but not medical personnel)											
No	4,122	15.5%	<0.05	16.4%	<0.001	11.1%	<0.001	10.0%	<0.001	39.2%	<0.001
Yes	884	18.8%		26.6%		20.2%		19.8%		52.1%	
Among the medical staff											
No	3,868	14.6%	<0.001	16.6%	<0.001	11.0%	<0.001	9.6%	<0.001	38.8%	<0.001
Yes	1,138	21.1%		23.4%		18.5%		18.9%		50.5%	
In traditional media (press, radio, television)											
No	4,413	15.1%	<0.001	16.8%	<0.001	11.5%	<0.001	10.5%	<0.001	39.1%	<0.001
Yes	593	23.2%		28.1%		21.7%		20.7%		58.8%	
In scientific journals											
No	4,540	15.2%	<0.001	16.8%	<0.001	11.2%	<0.001	10.4%	<0.001	39.2%	<0.001
Yes	466	24.4%		31.6%		27.8%		25.1%		63.6%	

The multivariable logistic regression model predicting the implementation of personal interventions to reduce air pollution exposure obtained the Cox and Snell *R*-square of 0.123, and the Nagelkerke R-square of 0.166 (Table 5). Males had a 15% higher odds of using ≥1 intervention (OR = 1.15; 95%CI: 1.02–1.31). Those aged 25–34 had 48% higher odds compared to those aged 45–64 (OR = 1.48; 95%CI: 1.24–1.78). No statistically significant association was observed for educational level. Residents of the largest cities (>500,000 inhabitants) had 46% higher odds compared with rural residents (OR = 1.46; 95%CI: 1.19–1.78). Having children up to 4 years (OR = 1.24; 95%CI: 1.02–1.51) and 5–12 years (OR = 1.24; 95%CI: 1.06–1.44) also increased the odds of implementation of personal intervention relative to those without children in these age groups. Individuals with frequent infections (≥5 times/year) had 46% higher odds (OR = 1.46; 95%CI: 1.07–2.00) than those who reported not getting sick at all. No association was found with the number of chronic diseases. Those who do not seek health information had 60% lower odds (OR = 0.40; 95% CI: 0.34–0.47). Conversely, seeking information on public institutions’ websites was associated with 63% higher odds (OR = 1.63; 95% CI: 1.33–1.99) to implement personal interventions to reduce air pollution in comparison to people who declared that they did not get sick at all. Detailed data are presented in Table 5.

**Table 5:** Multivariable logistic regression model predicting the implementation of ≥1 personal intervention to reduce air pollution exposure.

	*p*	OR (95%CI)
Gender		
Male	<0.05	1.15 (1.02–1.31)
Female	Ref.	Ref.
Age		
18–24	<0.05	1.29 (1.03–1.61)
25–34	<0.001	1.48 (1.24–1.78)
35–44	<0.01	1.32 (1.12–1.55)
45–64	Ref.	Ref.
Educational level		
Primary or vocational	Ref.	Ref.
Secondary	0.176	0.9 (0.77–1.05)
Higher	0.925	1.01 (0.85–1.2)
Place of residence		
Rural area	Ref.	Ref.
City < 100,000 residents	<0.001	1.39 (1.2–1.6)
City 100,000–499,000 residents	<0.001	1.5 (1.25–1.79)
City ≥ 500,000 residents	<0.001	1.46 (1.19–1.78)
Having children under 4 years of age		
No	Ref.	Ref.
Yes	<0.05	1.24 (1.02–1.51)
Having children aged 5–12 years		
No	Ref.	Ref.
Yes	<0.01	1.24 (1.06–1.44)
Having children aged 13–17 years		
No	Ref.	Ref.
Yes	0.393	0.93 (0.79–1.09)
Having job (currently working)		
Yes, full-time job	0.145	1.13 (0.96–1.32)
Yes, part-time job	<0.001	1.73 (1.43–2.08)
No	Ref.	Ref.
Self-assessment of household financial situation		
We have enough money for everything and we are still saving for the future	<0.001	1.62 (1.27–2.06)
We have enough money for everything without any special sacrifices but we do not save for the future	<0.01	1.45 (1.14–1.85)
We live frugally and thanks to that we have enough money for everything	0.115	1.19 (0.96–1.49)
We live very frugally in order to save money for more serious purchases	<0.05	1.36 (1.05–1.75)
We only have enough money for basic needs or there is not enough money even for the cheapest food	Ref.	Ref.
On average. How often do you get sick or catch a cold during the year?		
I never get sick	Ref.	Ref.
1–2 times a year	0.539	1.07 (0.86–1.34)
3–4 times a year	<0.01	1.41 (1.1–1.82)
5 times a year or more	<0.05	1.46 (1.07–2)
Number of chronic diseases		
No disease	Ref.	Ref.
1 disease	0.215	1.1 (0.95–1.28)
2 diseases	0.902	0.99 (0.82–1.19)
3 or more	0.113	1.17 (0.96–1.43)
I am not looking for health information		
No	Ref.	Ref.
Yes	<0.001	0.4 (0.34–0.47)
On the websites of public institutions (e.g., Ministry of Health or WHO)		
No	Ref.	Ref.
Yes	<0.001	1.63 (1.33–1.99)
On social media (e.g., Facebook, Instagram, TiKTok)		
No	Ref.	Ref.
Yes	<0.01	1.29 (1.1–1.52)
In traditional media (press, radio, television)		
No	Ref.	Ref.
Yes	<0.001	1.41 (1.16–1.7)
In scientific journals		
No	Ref.	Ref.
Yes	<0.001	1.63 (1.32–2.02)

## 4 Discussion

This secondary analysis provides a comprehensive characteristics of personal intervention to reduce air pollution exposure among adults aged 18–64 years in Poland. This study revealed that a low percentage of Poles have implemented a personal intervention to reduce air pollution exposure. Closing windows on highly polluted days was most common (18.2%), whereas only 11.8% reported monitoring air quality alerts. Significant socio-demographic differences in the implementation of personal intervention to reduce air pollution exposure were identified. Male gender, younger age, living in urban areas, having children aged 4–12 years, having a part-time job, good household economic situation, ≥3 infections per year, and seeking health information on official websites of public institutions, social media, traditional media, and scientific journals were associated (*p* < 0.05) with implementing ≥1 personal intervention to reduce air pollution exposure.

Several systematic reviews and meta-analyses pointed out difficulties in assessing effectiveness of individual measures to reduce air pollution exposure (12, 18, 19). Burns et al. (18) concluded that, due to heterogeneity across interventions, outcomes, and methods, it was difficult to derive overall conclusions regarding the effectiveness of interventions in terms of improved air quality or health. Janjua et al. (12) stated that the lack of evidence and study diversity has limited the conclusions of their review. Hlophe et al. (19) also pointed out some gaps in current research.

American Thoracic Society (ATS) recommends the following several measures to individuals to reduce their exposure and risk of adverse health effects, including staying indoors, limiting physical activity during times with elevated pollution concentrations and near air pollution sources, cleaning indoor air with central system filters or portable room air purifiers, and using personal protective devices such as respirators (20). The ATS also underscores that key barriers to personal interventions include affordability and the difficulty of communicating complex behavior changes in ways that fit patients’ priorities, circumstances, and values (20). Environmental justice and equity should be integral to any recommendations. Guideline development should proceed in parallel with assessments of information accessibility, the availability of financial support, and practical mechanisms to help individuals afford and sustain these interventions (20). In this study, barriers to personal interventions were not analyzed.

Air pollution – especially in urban areas – is a significant public health problem in Poland (14, 21). Air quality standards are frequently exceeded, particularly in southern regions and during winter (21). Southern Poland’s reliance on solid fuels in old household boilers—major “low-stack” emitters of PM and benzo(a)pyrene—drives winter smog. Valleys, basins, and temperature inversions trap these pollutants, while historic industry and dense traffic (e.g., Upper Silesia, Kraków) add to the burden. Nevertheless, discussions on environmental health, air pollution, and climate change are often limited or neglected in public discourse (14). Coal remains a significant domestic energy source. For historical and social reasons, debate on air pollution and environmental health in Poland has been constrained (22). To date, there was a lack of nationwide campaigns on personal interventions to reduce air pollution exposure. Available campaigns have primarily focused on financial relief and subsidies for replacing household heating systems. Therefore, findings from this study provide important data from an EU country, with relatively high exposure and limited educational activities on personal protection.

Across the EU, air-quality data are widely available through the websites of the European Environmental Agency, national institutions, and private monitoring platforms (23, 24). Air quality monitoring systems are typically free, user-friendly, and provide simple traffic-light indicators (green for good air quality, yellow for moderate, and red for bad air quality) (25). In this study, only 11.8% of adults reported monitoring alerts. This observation suggesting limited engagement despite wide availability of air-quality data.

Closing windows during polluted periods is a simple, no-cost intervention that can limit infiltration (26). Although its effect on exposure may be modest, it is easy to implement in response to rising pollutant levels (10–12). In this study, closing windows in polluted seasons was the most common personal intervention to reduce air pollution exposure. In Poland, household heating is a major emission source; some households also burn waste for economic reasons. We can hypothesize that odors associated with the burning of garbage and materials such as plastic or tires may prompt window closing. Due to the relatively rare use of air quality monitoring tools, such interventions like closing windows is likely triggered by organoleptic cues, reducing effectiveness for odorless pollutants.

During physical exercise, the inhaled dose of air pollutants increases (27). It is believed that air pollution can attenuate the cardioprotective effects of physical activity, so outdoor sports should be limited during high-pollution episodes (28, 29). It is recommended to do walking, running, or cycling in low-traffic areas, to reduce exposure (28, 29). In this study, 12.7% reported avoiding outdoor walking during high pollution. Such a low percentage of individuals who declared this intervention may reflect the high frequency of polluted days in winter, making avoidance difficult and potentially leading to risk normalization.

Portable air purifiers can reduce indoor particulate matter in the short term and may confer cardiovascular and respiratory benefits (30–32). Contrary to previously described interventions (air quality monitoring, closing windows, and limiting outdoor activities), purifiers require attention and financial outlays (purchase and filter replacement). In this study, only 16.1% reported using air purifiers all year round or during the heating season. This observation showed low implementation of air purifiers in households in Poland and may reflect both limited awareness and economic barriers (device cost and, in larger apartments, the need for multiple units). Using air purifier is an effective measure to protect from air pollution exposure. Brugge et al. (33) showed that the use of in-home high-efficiency particulate arrestance (HEPA) air purifiers resulted in clinically important reductions in systolic blood pressure for people with elevated systolic blood pressure in environments with relatively low PM_2.5_ concentrations. Singh-Smith et al. showed that near highway residents can be receptive and adherent to using portable air purifiers in homes on a regular basis (34). Further educational activities are needed in Poland to strengthen public awareness or air purifiers and their impact on human health.

In this study, the multivariable logistic regression model was prepared to identify factors associated with the implementation of ≥1 intervention. Males were more likely to implement personal intervention to reduce air pollution exposure. In a previously published nationwide cross-sectional study on digital health technologies and wearables in Poland, there were no gender differences in the prevalence of the use of different e-health technologies (35). This observation requires further investigation. Individuals aged 18–44 years were more likely to implement personal intervention to reduce air pollution exposure than those aged 45–64 years. This observation may result from the higher knowledge of environmental health and the perception of climate as a serious threat to younger generations (36). Those who lived in urban areas were more likely to implement ≥1 intervention. Inhabitants of urban areas are more exposed to air pollution than those from rural areas (37, 38). Transportation, motor vehicles and urban traffic significantly contribute to higher exposure to air pollution in cities (39). Due to the higher exposure, inhabitants of cities may be more likely to implement personal intervention to reduce air pollution (39). Having children aged 4–12 years was also associated with higher odds of implementing a personal intervention to reduce air pollution exposure. Small children are particularly vulnerable to air pollution, especially those with asthma and allergies (40). We can hypothesize that parents are implementing interventions to reduce air pollution exposure to protect their children from the adverse health effects of air pollution exposure. This study is based on data from a representative sample of adults of working age (18–64 years). Those who had part-time jobs were more likely to implement personal interventions to reduce air pollution. This may result from the fact that they spend more time at home than those with full-time jobs, so they may more often experience exposure to air pollution. People with low socioeconomic status are at higher risk for exposure to air pollution (41). In this study, a good household economic situation was associated with the implementation of ≥1 intervention. This observation is in line with the general thinking that wealthy people are more likely to care about the environment and limit exposure to environmental health hazards (42, 43).

Exposure to particulate matter and traffic-related pollutants is associated with increased risk of respiratory infections (44). In this study, respondents reporting better health than peers were more likely to take protective actions, but those with frequent infections (≥3–5/year) were also more likely to do so, suggesting problem-driven prevention (seeking cleaner air to mitigate symptoms) (44). In this study, there was no association between the number of chronic diseases and intervention uptake, highlighting potential gaps in environmental health education for patients with respiratory and cardiovascular conditions (45).

Overall, interest in health information positively correlated with protective behaviors—even information obtained via social media increased the likelihood of using exposure-reduction methods. This underscore the importance of digital environmental health education (46).

There is a lack of nationwide data on air pollution protection in Poland. A similar study on environmental hazard protection in Poland showed that 65.0% of adults aged 18–64 years had implemented ≥1 intervention to reduce exposure to nighttime light pollution, wherein the use of blackout curtains in the bedroom was the most common intervention (47).

### 4.1 Practical implications

Poland’s exceedances are driven by solid-fuel heating and topography/meteorology, especially in the south. This study has practical implications for public authorities and public health specialists. This study revealed serious gaps in the implementation of personal intervention to reduce air pollution in Poland. Gender, age, place of residence, having children and economic status were significant socio-demographic factors that were associated with public attitudes toward the implementation of personal intervention to reduce air pollution, and these differences should be considered when planning public health intervention on environmental health. Moreover, this study underlined the role of environmental health education in official websites of public institutions as well as social media. Rural–urban and economic disparities in the implementation of personal intervention to reduce air pollution seems to be a serious health inequalities that requires adequate actions planned in public health strategies. Poland’s exceedances are driven by solid-fuel heating and topography/meteorology, especially in the south.

### 4.2 Limitations

This is a secondary data analysis, and the original datasets came from a cross-sectional survey conducted with computer-assisted web interviews, which is the major limitation of this study. The scope of analysis was limited to the data available in the original dataset, acquired for this analysis. In this study, only four personal interventions to reduce air pollution exposure were analyzed. Data on air quality in the homeplace was not collected, and indoor air pollution levels were not measured. Data were self-reported and recall bias might occur. Obtaining more accurate data (observation of individual behavior) is very difficult, especially on such a large group.

## 5 Conclusion

This study revealed substantial gaps in the implementation of personal exposure-reduction measures among adults in Poland, a country with a high burden of air pollution. Socio-demographic differences in the implementation of a personal intervention to reduce air pollution were observed, with particular emphasis on gender, age, place of residence, having children, and economic status. Given the continuing public health threat posed by air pollution, additional educational and legislative actions are needed to protect the Polish population. Particular emphasis should be paid to education on the health effects of air exposure (including short-term effects) to patients with chronic respiratory and cardiovascular diseases. More actions focused on strengthening health competencies related to environmental health and individual-level and household-level protection against air pollution are urgently needed.

## Data Availability

The data analyzed in this study is subject to the following licenses/restrictions: the data that support the findings of this study are available from the National Centre for Health Policy and Health Inequalities of the Cardinal Stefan Wyszynski University but restrictions apply to the availability of these data, which were used under license for the current study, and so are not publicly available. Data are however available from the authors upon reasonable request and with permission of the National Centre for Health Policy and Health Inequalities of the Cardinal Stefan Wyszynski University. Requests to access these datasets should be directed to https://ncpz.uksw.edu.pl/dzialalnosc/.
