# Supplementary material for: Personal interventions to reduce air pollution exposure in a representative sample of Poles aged 18–64 years
Source: Front Public Health. 2025 Nov 26;13:1656587. doi: 10.3389/fpubh.2025.1656587 (PMC12689963; doi:10.3389/fpubh.2025.1656587)
Supplement: Supplementary file 1 [file Data_Sheet_1.PDF]

P1. Respondent's gender:

a) Male
b) Female

P2. In what year were you born? _ _ _ _

P3. What is your education level? Please provide the highest level of education you have achieved.

a) rural area
b) city below 100,000 residents
c) city 100,000-499,000 residents
d) city >=500,000 residents

P4. What is your education level? Please provide the highest level of education you have achieved.
e) Unfinished primary or no school education
f) Primary
g) Lower secondary
h) Basic vocational (also SPR)
i) General secondary without matriculation exam
j) General secondary with matriculation exam
k) Vocational secondary without matriculation exam
l) Vocational secondary with matriculation exam (technical school, vocational or technical high school)
m) Post-secondary or post-secondary
n) Higher education with the title of engineer, bachelor, certified economist
o) Higher education with a master's degree, medical degree or equivalent
p) Higher education with a doctoral degree or higher

P5. Are you currently gainfully employed (full-time, in your own company or farm or do you perform commissioned work)?
a) Yes, full-time
b) Yes, part-time
c) Yes, occasional
d) No

P6. How many people are in your family?
a) number of household members: |__|__|
including:
1. children: number of children under 4 |__|__|
2. children: number of children under 5-8 |__|__|
3. children: number of children under 9-12 |__|__|
4. number of children under 13-17 |__|__|
5. number of children 18 or older |__|__|

P7. Which of the following best describes the financial situation of your household?
a) We have enough for everything and we are also saving for the future
b) We have enough for everything without any special sacrifices but we are not saving for the future
c) We live frugally and thanks to this we have enough for everything
d) We live very frugally to save for more serious purchases
e) We only have enough money for basic needs
f) We do not have enough money even for the cheapest food

*P8. Which of the following personal interventions to reduce air pollution exposure do you make (MULTIPLE CHOICE)*
*a) I use air purifiers in my home all year round*
*b) I use air purifiers in my home during the heating season*
*c) I close windows to reduce air pollution (e.g. due to traffic or smog)*
*d) I avoid walking outside during periods of high air pollution*
*e) I monitor air quality alerts*
*f) None of the above*

*P9. Do you suffer from any of the following diseases? [YES/NO/I DON'T KNOW - for each]*
*a) Allergies (e.g. skin allergies, hay fever)*
*b) Food intolerance, food allergy*
*c) Urinary tract diseases (urolithiasis, kidney failure, glomerulonephritis, other kidney or urinary tract diseases)*
*d) Circulatory system diseases (hypertension, previous stroke, previous myocardial infarction, coronary artery disease, heart failure, atherosclerosis of the lower limb arteries, lipid disorders (high: total cholesterol, LDL, triglycerides), other heart diseases)*
*e) Musculoskeletal diseases (joint disease, osteoporosis, other)*
*f) Type 1 diabetes*
*g) Type 2 diabetes*
*h) Digestive system diseases (peptic ulcer disease, gastroesophageal reflux disease, bowel disease, liver disease, pancreas disease, gallstone disease biliary, other)*
*i) Respiratory diseases (COPD, tuberculosis, asthma, other)*
*j) Endocrine diseases (hypothyroidism, hyperthyroidism, other)*
*k) Neurological diseases (Parkinson's disease, multiple sclerosis, other)*
*l) Cancer*
*m) Skin diseases (including psoriasis)*
*n) None of the above*

*P10. On average, how often during the year are you sick or have a cold, excluding situations resulting from a chronic disease)*
*a) I never get sick*
*b) 1-2 times a year*
*c) 3-4 times a year*
*d) 5-6 times a year*
*e) 7-12 times a year*
*f) More than 12 times a year*

*P11. Where do you look for information on health-related topics [multiple choices]*
*a) I am not looking for information on health*
*b) On the websites of public institutions (e.g. WHO, GIS, MZ)*
*c) On the websites of health services (e.g. medonet, abczdrowie, poradnikzdrowie)*
*d) On other websites or internet forums*
*e) On social media (e.g. Facebook, Instagram, TiKTok)*
*f) On YouTube or similar internet services*
*g) Among friends (but not medical personnel)*
*h) Among family members (but not medical personnel)*
*i) Among medical personnel*
*j) In traditional media (press, radio, television)*
*k) In scientific journals*

*P12. Is my health in comparison to people my age?*
*a) Definitely better*
*b) A little better*
*c) The same*
*d) A little worse*
*e) Definitely worse*
*f) Hard to say*
